# Bridging the Milk Gap: Integrating a Human Milk Bank–Blood Bank Model to Reinforce Lactation Support and Neonatal Care

**DOI:** 10.3390/nu17111765

**Published:** 2025-05-23

**Authors:** Jacqueline Barin, Jeremy Touati, Agathe Martin, Carole Fletgen Richard, Ralf J. Jox, Stefano Fontana, Hélène Legardeur, Nathalie Amiguet, Isabelle Henriot, Christelle Kaech, Aurélia Belat, Jean-François Tolsa, Michel Prudent, Céline J. Fischer Fumeaux

**Affiliations:** 1 Clinic of Neonatology, Department of Mother-Woman-Child, Lausanne University Hospital, University of Lausanne, 1011 Lausanne, Switzerland; jacqueline.barin@chuv.ch (J.B.); nathalie.amiguet@chuv.ch (N.A.); isabelle.henriot@chuv.ch (I.H.); jean-francois.tolsa@chuv.ch (J.-F.T.); 2 Interdisciplinary Perinatal Unit for Breastfeeding Support and Infant Nutrition, Department of Mother-Woman-Child, Lausanne University Hospital, University of Lausanne, 1011 Lausanne, Switzerland; 3 Laboratoire de Préparation Cellulaire et d’Analyses, Transfusion Interrégionale CRS, 1066 Epalinges, Switzerland; jeremy.touati@itransfusion.ch (J.T.); agathe.martin@itransfusion.ch (A.M.); aurelia.belat@itransfusion.ch (A.B.); michel.prudent@itransfusion.ch (M.P.); 4 Gynaecology-Obstetrics, Department of Mother-Woman-Child, Lausanne University Hospital, University of Lausanne, 1011 Lausanne, Switzerland; carole.richard@chuv.ch (C.F.R.); helene.legardeur@chuv.ch (H.L.); 5 Unité d’Éthique Clinique, Institut des Humanités en Médecine, Lausanne University Hospital, University of Lausanne, 1011 Lausanne, Switzerland; ralf.jox@chuv.ch; 6 Faculty of Biology and Medicine, University of Lausanne, 1011 Lausanne, Switzerland; stefano.fontana@itransfusion.ch; 7 Transfusion Interrégionale CRS, 3008 Bern, Switzerland; 8 HESAV School of Health Sciences, HES-SO University of Applied Sciences and Arts Western Switzerland, 1011 Lausanne, Switzerland; christelle.kaech@hesav.ch; 9 Laboratoire de Recherche sur les Produits Sanguins, Transfusion Interrégionale CRS, 1066 Epalinges, Switzerland; 10 Center for Research and Innovation in Clinical Pharmaceutical Sciences, Lausanne University Hospital, University of Lausanne, 1011 Lausanne, Switzerland; 11 Institute of Pharmaceutical Sciences of Western Switzerland, University of Geneva, 1205 Geneva, Switzerland

**Keywords:** human milk, human milk bank, *Lactarium*, donor human milk, transfusion, medical product of human origin, substance of human origin, breastfeeding, lactation, infant nutrition, preterm infant, vulnerable neonate, Swiss health system

## Abstract

Mother’s own milk (MOM) offers the highest protection for preterm and low birth weight infants. However, breastfeeding can be challenging during neonatal hospitalization. When MOM is unavailable, donor human milk (DHM) is the recommended alternative for feeding vulnerable neonates. Human milk banks (HMBs) collect, process, and deliver DHM, playing a key role in lactation support and promoting MOM availability. Although HMBs are expanding globally, scale-up remains hindered, restricting equitable DHM access. In Switzerland, despite the existence of eight HMBs, the western region lacked such a facility until 2022. To address this gap, an interdisciplinary team from the Lausanne University Hospital (CHUV) and the Swiss Red Cross Interregional Blood Transfusion Centre (TIR) collaborated to establish a regional HMB. This partnership leveraged both institutions’ available expertise, infrastructure, and resources. After two years of preparation, the *CHUV Lactarium* launched in 2022 with the support of the Department of Health and Social Action (DSAS) of the Canton of Vaud. This novel *human milk bank–blood bank* model is fully integrated into the hospital’s neonatal care, nutrition, and breastfeeding programs, operating under a strict quality and coordination system. Since its implementation, the HMB has met 100% of DHM needs, with an 80% breastfeeding bridging rate. It has had a positive impact on neonatal care, family engagement, professional interest, and community awareness of human milk. This case study illustrates how synergistic collaboration can help bridge gaps in establishing a safe, efficient, and equitable HMB model. It also offers a scalable framework adaptable to other settings.

## 1. Introduction

Human milk is a uniquely complex and dynamic system of nutritional and biologically active components that promote optimal infant growth, immune protection, and development [1,2]. Moreover, breastfeeding provides short- and long-term health benefits for the mother–child dyad [3], offering significant public health, economic, and ecological advantages [4,5].

For vulnerable neonates—such as those born preterm, with low birth weight or with other medical issues—mother’s own milk (MOM) is especially critical due to its unparalleled protective properties, which reduce the risk of several complications and improve long-term outcomes [6,7]. While these infants have the highest need for MOM, they are less likely to initiate or sustain breastfeeding [8,9]. Multiple factors contribute to the lower prevalence and shorter duration of MOM feeding in preterm infants, including maternal comorbidities, complications, stress, lactation immaturity, impaired infant sucking, and healthcare organizational barriers, such as parent–infant separation [10]. While strong breastfeeding support and policies are essential, they often do not fully address all these obstacles [7,11].

In situations where the provision of MOM is delayed, insufficient, impossible, or undesired, donor human milk (DHM) is recommended as the second-best option for feeding preterm, low birth weight, or other at-risk newborns [3,12,13]. DHM is generallyprovided by lactating women who voluntarily donate their surplus milk to a human milk bank (HMB)—known in French as a ‘*Lactarium*’ [14,15,16]. Compared to formula, DHM halves the risk of necrotizing enterocolitis (NEC), a life-threatening condition affecting 5–15% of very preterm infants [17]. DHM also improves enteral feeding tolerance, shortens hospital stays, and lowers healthcare costs [18,19]. Additionally, DHM can serve as a temporary ‘bridge’ until the MOM supply becomes sufficient, positively supporting MOM provision and breastfeeding continuation [20,21]. Ensuring DHM safety generally involves HMBs overseeing donor recruitment and screening, along with milk collection, pasteurization, analysis, storage, and distribution [14,15,16]. Moreover, integrating HMBs into breastfeeding support and neonatal care programs is strongly recommended [22,23,24].

In 2017, the World Health Organization (WHO) classified DHM as a ‘medical product of human origin’ (MPHO) [22], and in 2024, the European Union designateddonor human milk (DHM) as a ‘substance of human origin’ (SoHO) [23]. Despite these developments, the absence of global guidelines and harmonized legal frameworks still results in large operational heterogeneity among the approximately 700 HMBs worldwide [24,25,26]. Based on their structural organization, HMBs are categorized as centralized or decentralized, hospital-based or community-based, and independent or integrated within maternal and neonatal care systems [14]. Most HMBs focus solely on DHM production, but hybrid models combining milk banking with blood or tissue banking also exist in several countries (notably Australia, Canada, USA, Spain, and Germany) [27,28]. However, these models vary, and data on such ‘human milk bank–blood bank’ systems remain scarce.

Although the number of HMBs is growing globally, their distribution remains uneven and insufficient to meet DHM needs in many settings. While further scale-up is essential, HMBs still face multiple implementation barriers [25]. On the other hand, the rise of for-profit companies commercializing human milk-derived products in unregulated markets raises ethical concerns [29,30]. Global guidance is therefore needed to support the ethical, equitable, and sustainable development of HMBs [25,26,31]. Meanwhile, marked inequities in DHM access persist both between and within countries [24], as illustrated by the case of Switzerland [32,33].

**Figure 1:**
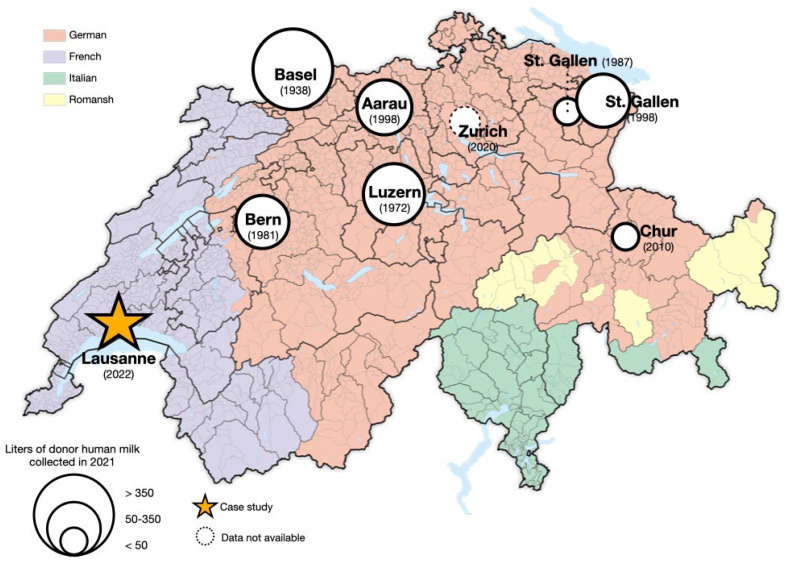
Human milk banks in Switzerland (year of establishment) and volumes of donor human milk collected in 2021. Map source: Swiss Federal Statistical Office.

Switzerland is a multilingual and multicultural country in central Europe, comprising of 26 ‘cantons’ (states) and four linguistic regions (German, French, Italian, and Romansh). Its healthcare system is decentralized and primarily governed at the cantonal level, which oversees hospitals and public health policy. Even in a high-resource country with established national guidelines [34], access to DHM for vulnerable infants varies considerably, with marked regional disparities [32,33]. As shown in Figure 1, prior to 2022, Switzerland had eight hospital-based HMBs of varying sizes—none of which served the French- or Italian-speaking regions. The absence of a HMB in the French-speaking region denied DHM access to over 25% of the roughly 80,000 infants born annually in Switzerland, including 6–7% preterm and 1–2% very preterm [35]. The Italian-speaking region accounts for only 3% of births, and very preterm or very low birth weight infants from this area are transferred to level III neonatal units in other regions, making the lack of a HMB in this area less critical.

Facing this gap, the Swiss government encouraged the scale-up of HMBs in 2019 [36]. However, the absence of a defined regulatory framework, consistent policies, funding models, or health insurance reimbursement has continued to hinder such development. The lack of national coordination has also perpetuated a fragmented network of local HMBs, each primarily serving its hospital, with limited scope for broader collaboration.

To address the lack of access to DHM in western Switzerland, an interdisciplinary team, united by a common goal to establish a human milk bank, brought together neonatology, nutrition, and lactation specialists from Lausanne University Hospital (CHUV) alongside blood banking experts from the Interregional Blood Transfusion (TIR) establishment of the Swiss Red Cross. The collaborative team adopted a stepwise project approach to design and implement a HMB model specifically tailored to address contextual barriers while adhering to international safety and quality standards.

In this case study, we describe the implementation of this HMB model designed to:

i. Ensure the safe, efficient, equitable, and sustainable provision of DHM to meet the regional needs of patients.
ii. Complement and strengthen the CHUV’s neonatal care, infant nutrition, and lactation support programs.

## 2. Methods

### 2.1. Design

This case study reports the project partnership, development phases, barriers, facilitators, implementation process, and initial impacts of a novel HMB in the French-speaking region of Switzerland.

### 2.2. Partnership Project Setting

The Lausanne University Hospital (CHUV), located in the canton of Vaud in western Switzerland, serves as a Level III perinatal center and referral hospital for over 16,000 births annually across four French-speaking cantons. It features a 40-bed neonatology unit, one of the largest in Switzerland, which admits approximately 900 neonates each year, half of whom are born preterm. In 2016, CHUV established a breastfeeding support unit for hospitalized neonates, which provides lactation consultations, staff training, and peer-to-peer counselling. This program significantly improved breastfeeding outcomes and laid the groundwork for quantifying annual DHM needs as well as evaluating potential sources [11].

The Interregional Blood Transfusion (TIR) establishment of the Swiss Red Cross is a non-profit organization that supplies hospitals in the cantons of Vaud, Bern, and Valais with blood products and provides services in transfusion medicine. TIR has strong expertise in blood donation, biosafety, quality assurance, research, and innovation.

Prior to the HMB project, CHUV and TIR already had a partnership centered around blood-derived products. Both institutions recognized a strategic interest in establishing a regional HMB and that their collaboration would optimize the use of complementary expertise, infrastructure, and resources, thereby enhancing feasibility within reasonable timelines. For instance, CHUV could resolve hospital infrastructural constraints by utilizing suitable facilities available at TIR. The two institutions are located less than 3 km apart and are connected by a direct metro line, along with a daily transport of blood products.

### 2.3. Project Team and Management

The interprofessional, interdisciplinary team from both partner institutions led the project, brought together by their shared commitment to developing a regional HMB. They leveraged the complementary strengths of TIR in biosafety, ethical donation frameworks, and quality assurance, alongside CHUV’s clinical expertise in neonatology, nutrition, and lactation care. The panel consisted of clinicians (pediatricians, obstetricians, midwives, lactation consultants, and nurses), biologists, and specialists in engineering, hygiene, ethics, quality management, finance, law, and communication, who collaborated across three organizational levels: a steering committee, a core team, and eight working groups.

The team used a project management software (Antura© version 23.9), which facilitated the coordination of objectives, deliverables, timelines, responsibilities, budgeting, and risk analysis. The project had four key phases:

(1) *Baseline needs assessment and model identification:* This initial stage aimed to (i) quantify DHM needs using breastfeeding unit monitoring data, (ii) identify the most suitable HMB model through literature review, stakeholder consultations, on-site visits, and interdisciplinary expert meetings, and (iii) confirm the proposed solution via discussions with policymakers.
(2) *Development and preparation:* This phase focused on (i) defining partners’ roles and responsibilities for future operations, (ii) preparing documentation, protocols, and workflows, and (iii) procuring, analyzing, and qualifying the necessary equipment. These tasks were performed by joint working groups and overseen by the steering committee.
(3) *Launch:* This phase centered on (i) operationalizing workflows, (ii) monitoring key performance indicators, and (iii) adjusting processes based on early outcomes.
(4) *Operational and monitoring phase:* This phase intended to (i) consolidate practices, (ii) conduct regular reviews involving the operational group, steering committee, institutional leaders, and public health authorities, and (iii) optimize processes through continuous data monitoring.

### 2.4. Continuous Data Monitoring

To facilitate ongoing improvement and ensure alignment with the project objectives, data registries were developed using Microsoft Office Excel© (v16.77) and REDCap© (v15.0.12) to systematically collect and monitor key operational and clinical indicators, which include:

- Human milk volumetry (volumes of human milk collected, pasteurized, analyzed, qualified, delivered, administered, discarded).
- Donor information (demographic data, medical and nutritional histories, lifestyle factors, serology results, documented informed consent).
- Recipient patient data (demographics, medical indications for DHM, parental consent).
- Clinical outcomes (neonatal mortality rates, associated complications, and duration of hospital stays).
- Nutrition and breastfeeding outcomes (duration of enteral and parenteral nutrition, volumes of MOM and DHM and/or formula intake during hospitalization, as well as breastfeeding rates recorded at initiation and discharge).

Process safety and traceability were ensured by the following systems:

- A dedicated human milk banking module integrated into the TIR TCS v1.1 software by Mak-System©, which facilitates real-time monitoring and guarantees traceability, quality, and safety across all operational steps.
- CHUV’s lacto-vigilance software (Almalacté© v1.24.1.0) ensures the safety and full traceability of milk from the medical prescription and ordering to its administration to the patient.
- CHUV’s electronic patient medical record system (Soarian© v4.7.100 and Metavision© v6.14).
- Swiss Minimal Neonatal Data Set, which collects the benchmarking data of neonates born between ≥22 and <32 weeks gestational age or with birth weight between 400 g and 1500 g in Swiss Level III and IIb neonatal care centers.

### 2.5. Ethics

As a quality assurance initiative, this HMB implementation case study was exempt from the Swiss Human Research Act and did not require approval from an ethics committee. Human milk donors sign an informed consent for the donation and potential biobanking if the milk is considered unsuitable for clinical use. Similarly, recipient parents are informed and must sign a consent form before their child receives DHM.

## 3. Results

### 3.1. Phase 1: Baseline Needs Assessment and Model Identification—2020

During the initial phase, clinical and nutritional data from the breastfeeding monitoring system were analyzed, allowing for the extrapolation of the required DHM volumes based on the formula intake of hospitalized neonates born at <32 weeks postmenstrual age or with a birth weight <1500 g, between 1 January 2019 and 31 December 2020. This assessment resulted in an estimated annual demand of 250 to 350 litres of DHM for approximately 200 patients.

In parallel, the team conducted a comprehensive review of international and national recommendations, guidelines, directives, case studies, and scientific literature related to human milk banking. This was complemented by expert interviews and on-site visits to several HMBs in Switzerland and abroad, including organizations operating as “human milk bank–blood banks” (e.g., Banc de Sang i Teixits© in Barcelona, Spain, Héma-Québec© in Montreal, Canada). Consultations were also held notably with members of the Swiss HMB group, the French Human Milk Bank Association (ADLF), the European Milk Bank Association (EMBA), and the Global Alliance of Milk Banks and Associations (GAMBA).

Based on these initial findings, the integration of a “human milk bank–blood bank” at CHUV, in collaboration with TIR, emerged as the most appropriate and sustainable solution. This approach ensures safe, efficient, and ethical access to DHM for vulnerable neonates while also complementing and enhancing the hospital’s existing initiatives in infant nutrition and breastfeeding support. This synergistic approach offers several key advantages in this setting, notably:

- *Leveraging complementary expertise* of the CHUV in neonatal care, nutrition, and lactation support and the TIR’s expertise in donation management, quality-controlled processing, and distribution of MPHOs.
- *Mutualizing existing infrastructures and resources*, such as the breastfeeding rooms at CHUV, the TIR laboratories, and serological testing services, alongside the integration and adaptation of IT systems and the use of existing logistics and transport networks between the CHUV and TIR facilitated the development of processes. The TIR’s regional transport system enabled the collection of milk directly from the donors’ homes, a strategy shown to enhance the sustainability of human milk donation [37]. Moreover, merging these infrastructures and services significantly reduced initial investment costs.
- *Safety and quality assurance*: HMB operations align with a rigorous, ethical, quality, and safety framework, based on TIR’s standards for human blood product processing and donor management, fully compliant with good manufacturing practices (GMP).
- *Regulatory perspectives*: the project incorporated considerations of evolving frameworks, particularly the ongoing European harmonization of human milk banking under the SoHO framework [23].

Upon completion of the initial phase, the Department of Health and Social Action (DSAS) of the Canton of Vaud formally confirmed its support for the HMB implementation, marking a decisive milestone in the establishment of this nonprofit initiative.

### 3.2. Phase 2: Project Development and Preparation—2021

**Figure 2:**
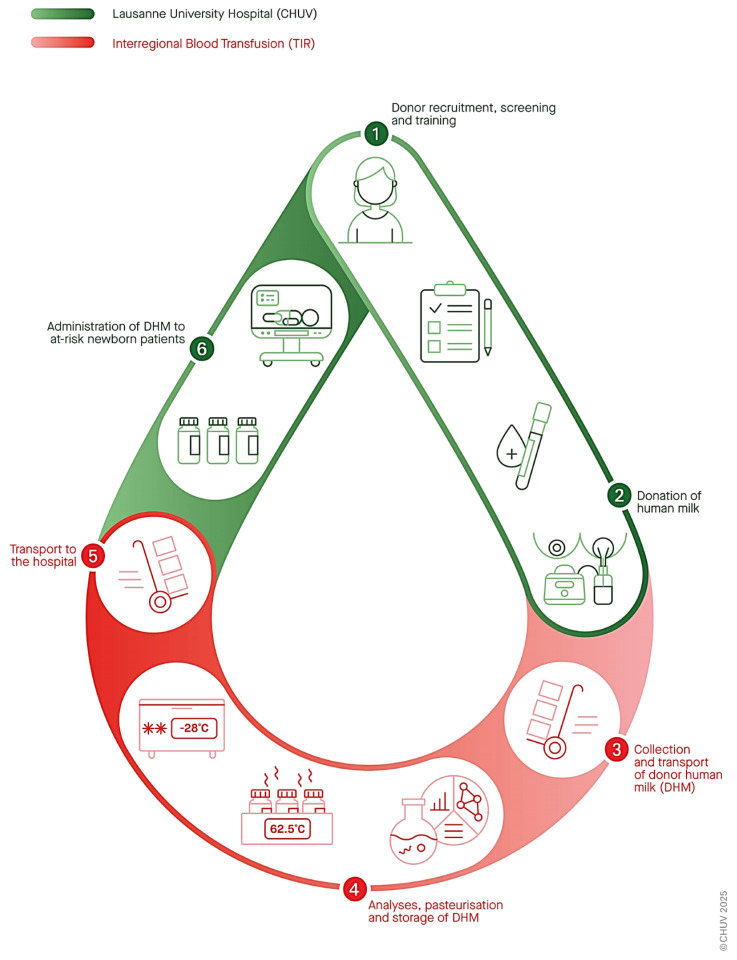
Circuit of donor human milk and CHUV-TIR partnership organization of the *CHUV Lactarium*.

Figure 2 illustrates the organizational structure of the HMB partnership across two sites and delineates the respective responsibilities, as outlined in detail by a formal agreement. Additional details on the processes are provided in the Supplementary Materials.

By 2021, the core team completed the project proposal, which was subsequently approved by the steering committee. Representatives from family associations were consulted during this stage. Multidisciplinary expert groups were then established to address key areas, including processing, microbiology, nutrition, logistics, ethics, recruitment, communication, IT, legal, and finance. Each group developed objectives, roadmaps, deliverables, and timelines, and conducted risk assessments. Standard operating procedures (SOPs) were prepared in accordance with GMP standards and validated by quality management specialists. Following a combination of national and international recommendations, the HMB system established specific processes for:

- Donor recruitment, screening, and training.
- DHM collection and transport.
- Processing (including multi-donor pooling, microbiological analyses, and Holder pasteurization).
- Storage (cold room at −30 °C for up to 6 months after pasteurization).
- Transport to the hospital (based on clinical needs).
- Administration to at-risk hospitalized neonates (following medical prescription and parental consent).
- Full process traceability from donors to recipients while maintaining anonymity.

Following operational planning, infrastructure and logistics were optimized, equipment and materials were procured and qualified, and software systems were customized to ensure process monitoring and DHM traceability. In parallel, staff training programs were initiated across both institutions. Communication efforts were also launched, including the development of a website to inform and raise awareness among the wider public (https://www.chuv.ch/fr/lactarium/). Finally, a pilot phase simulated the complete process, allowing for workflow validation, process optimization, and confirmation of implementation readiness.

### 3.3. Phase 3: Launch–2022

- *Launch:* In May 2022, after more than two years of strategic planning by the interdisciplinary team, the “*CHUV Lactarium*” was launched as a nonprofit, regional, “human milk bank–blood bank”. Integrated into neonatal care, it complemented the hospital’s nutrition and lactation support program. Funded by the government as a public health service, the initiative was well received by healthcare professionals, the public, and the media.

### 3.4. Phase 4: Operation and Monitoring—Ongoing Since 2022

- CHUV and TIR have collaborated closely to streamline their processes. Members from both institutions participate in (a) an operational team; (b) several advisory expert groups that provide periodic guidance on specific topics such as microbiology, quality management and bioethics; and (c) a monitoring committee that conducts biannual reviews to ensure oversight and enhance program strategies.
  At the core of this coordination, the operational team meets weekly to monitor and assess key performance indicators. DHM volumes are adjusted according to patients’ real-time needs to prevent shortages or waste. This is achieved through incremental donor recruitment strategies and a flexible schedule in DHM pasteurization. When necessary, DHM allocation follows a pragmatic, evidence-based prioritization system based on clinical benefit, adapted from French guidelines and other established practices [16,38]. Accordingly, three levels of prioritization are defined:
  - *Level 1*: In the event of a crisis or shortage, DHM provision would be restricted to the most vulnerable patients, i.e., those under 28 weeks postmenstrual age or weighing less than 1000g. Such restrictions have not been required to date.
  - *Level 2:* The standard target level, including all infants under 32 weeks postmenstrual age or weighing less than 1500 g.
  - *Level 3:* In the case of a temporary surplus, DHM may be administered to patients up to 34 weeks postmenstrual age or weighing up to 1800 g.

Moreover, critical medical conditions, such as therapeutic hypothermia, complex cardiopathies, or specific digestive malformations and surgical conditions, may justify individual considerations for DHM.

Since 2023, the core team has integrated “*Lacto-biobank*” services in response to a significant increase in human milk donation offers, which have surpassed the clinical need for DHM. The “*Lacto-biobank*” enables the preservation and storage of human milk that is not suitable for clinical use, thereby preventing it from being discarded and making it available for research and development purposes. The biobank adheres to the ethical standards established by the Swiss Biobanking Platform [39].

- *Monitoring and main achievements:* From May 2022 to December 2024, the *CHUV Lactarium* met 100% of the needs for DHM for eligible patients, resulting in a positive clinical impact. Key indicators and achievements are listed in Table 1.

## 4. Discussion

This case study illustrates how a synergistic partnership, combined with structured, phased project planning, can overcome common challenges and barriers to HMB implementation. It demonstrates the feasibility and effectiveness of an innovative operational model within the Swiss context, while offering promising opportunities for adaptation and scalability in other settings.

Launched in 2022, after 2 years of preparation, the *CHUV Lactarium* bridged the “milk gap” in western Switzerland through a pioneering “human milk–blood bank” organization. This nonprofit, publicly funded service successfully fulfilled its core objectives, ensuring safe, equitable, and sustainable access to donor human milk for vulnerable neonates in the region. As the first “human milk–blood bank model” in Switzerland, it is now among the country’s largest. Although published data on such hybrid models remain limited, similar organizations already exist in other countries, including Germany, Spain, Australia, Canada, and the United States [27]. Unlike other “human milk bank–blood bank” models, the *CHUV Lactarium* is uniquely embedded within neonatal care, nutrition, and lactation services—operating as a patient-centered, integrated HMB rather than an independent, product-oriented service [40]. Since its implementation, preliminary indicators suggest a positive impact on breastfeeding and clinical outcomes, consistent with findings reported in the literature [17,18,20].

### 4.1. Barriers and Facilitators

Despite the existence of Swiss HMB guidelines [34], the lack of regulatory and financial frameworks, in addition to national HMB and healthcare coordination systems were significant barriers in establishing a HMB in our context. Moreover, breastfeeding rates in western Switzerland are comparatively lower than in the rest of the country, suggesting a less ingrained culture of breastfeeding support in the region [41]. The project’s preliminary phase (2020–2022) coincided with the COVID-19 pandemic, which imposed further constraints on planning and implementation efforts.

Nonetheless, several key factors were instrumental in the successful establishment of the HMB:

- Interprofessional and interdisciplinary collaboration that facilitated effective coordination among partners and institutions.
- Leveraging complementary expertise, infrastructure, and resources.
- Pre-existing hospital-based breastfeeding support program, which served as a foundational “pillar”.
- Systematic preparation and phased project management.
- Robust joint leadership and endorsement from public health authorities.
- National and international HMB networking and mentorship.
- Active community outreach and media involvement.

### 4.2. Strengths and Interest

- *Case studies of novel HMB implementation*: Global HMB expansion is crucial to addressing disparities in accessing human milk [14,25]. Sharing and disseminating practical examples and analysis of scalable, efficient HMB implementations can support the scaling-up of HMBs across diverse resource settings [42,43,44,45].
- *Transferability*: Although Switzerland is a small, high-income country, its complex, decentralized healthcare system and lack of national coordination in human milk banking reflect challenges common to many settings. Its multilingual, multicultural context further exemplifies these difficulties. For instance, in Europe, despite over 280 operational HMBs and established guidelines [15], only 9 of 26 countries legally regulate DHM [46], while, in another survey, 7 of 10 lack reimbursement frameworks [47].
- *Leveraging established quality frameworks:* Integrating human milk banking within established blood and tissue donation frameworks facilitates the adoption of rigorous strategies, such as quality assurance and good manufacturing practices, thereby promoting high standards of quality and safety. This approach is likely to gain importance under forthcoming European regulations classifying human milk as a substance of human origin (SoHO) [23,48].
- *Enhancing breastfeeding:* Unlike other MPHOs, HMBs can reduce the demand for DHM by promoting breastfeeding and increasing MOM supply [28,49]. The establishment of HMB should reinforce, rather than replace, existing breastfeeding support strategies, viewing DHM as a bridge rather than a substitute for MOM. Therefore, integrated, patient-centered models that combine the provision of DHM with lactation support are more effective than independent, product-based approaches and are strongly recommended [14,50]. The *CHUV Lactarium* is among the first “human milk bank–blood bank” models embedded in neonatal care, infant nutrition, and lactation support.
- *Efficacy and equity:* This case study demonstrates that, even in a single-center setting, coordinated efforts and proactive planning for DHM needs can ensure consistent, efficient, and equitable distribution. This approach fully meets clinical demand without creating shortages or excess. Protocols were established to prioritize the most vulnerable infants during crises, though they have not yet needed to be activated [16,38,51].
- *Sustainability issues:* According to a recent review, MPHO-inspired HMB operational models help strengthen safety, enhance quality, and support regulatory-driven sustainability [28]. Additionally, DHM offers potential environmental benefits by supporting breastfeeding [20] and reducing formula use [52], aligning with the United Nations Sustainable Development Goals [53]. Although processing DHM can be considered an energy-intensive process, leveraging existing infrastructure (e.g., cold chambers, transportation, and IT systems) can help mitigate both financial and environmental costs. Currently, the *CHUV Lactarium* is pursuing actions to reduce plastic waste and collaborating in research to improve the sustainability of human milk donations [37].
- *Creation of the Lacto-biobank:* In compliance with Swiss Biobanking Platform standards, the *CHUV Lactarium* has operated a lacto-biobank since 2023 to redirect human milk unsuitable for clinical use towards research and development [39]. This strategy also helps to reduce donor frustration associated with milk rejection and enables the management of surplus donation proposals, which currently exceed patient DHM needs [54].

### 4.3. Limitations and Areas for Improvement and Development

- *Discarding rate*: Currently, approximately 20% of collected milk is discarded due to microbiological non-compliance, primarily identified from pre-pasteurization cultures of internal donors (i.e., mothers of hospitalized infants). Although this falls within the reported 7–27% range [55], it underscores challenges related to hygiene standards, testing protocols and microbiological criteria [56]. The operational team is actively addressing these issues to enhance process efficiency.
- *DHM volumes and scope for expansion:* The *CHUV Lactarium* currently supplies 250–300 L of DHM annually to a single center, ranking among the highest DHM volumes in Switzerland. Nevertheless, the DHM distribution volume and capacities remain limited compared to other, more centralized systems, such as in France or Canada. Scaling up production could improve cost-efficiency and help absorb the surplus of donations proposals, many of which are currently declined or redirected to the lacto-biobank. The *CHUV Lactarium* is therefore actively exploring options to expand its DHM distribution network.
- *Financial issues*: Although DHM provision is more expensive than formula [57,58], it has been shown to be cost-effective and even cost-saving, mainly by reducing the incidence of NEC and the associated healthcare costs [59]. However, the lack of financial models remains a common barrier to scale-up HMBs. Processing DHM as a MPHO may likely incur higher short-term costs than food-grade handling [50]. However, optimizing resources and increasing distribution volumes may help improve cost efficiency. A context specific medico-economic analysis, including long-term outcomes, is planned to support sustainable financial planning of the model.
- *Other perspectives and challenges:* As a recent HMB, the *CHUV Lactarium* needs to consolidate its expertise while adapting to numerous current and future developments in human milk science, processes, technologies, ethics, and regulations [47].

## 5. Conclusions

Despite the critical needs, significant gaps and paradoxes remain, thereby impeding safe, adequate, and equitable access to human milk for vulnerable infants. While HMBs are widely encouraged to increase the availability of both DHM and MOM, their implementation is often hindered by regulatory, financial, logistical, organizational, and political barriers. Therefore, practical case studies of successful HMB implementation, illustrating the application of universal HMB principles adapted to diverse contexts, can support and inspire stakeholders to overcome similar challenges.

This comprehensive report on the creation of the *CHUV Lactarium* highlights an innovative, patient-centered partnership between a tertiary hospital and a blood bank, resulting in the creation of western Switzerland’s first HMB. As a non-profit, government-funded project, it henceforth plays a critical role in improving equitable regional access to high-quality neonatal nutrition and care, strengthening breastfeeding support and raising public awareness. Its phased implementation underscores the importance of interdisciplinary efforts in bridging gaps and achieving strategic objectives. Such a collaborative approach shows promise in shaping future developments, research, and scalability.

## Figures and Tables

**Table 1:** Monitoring: key indicators and achievements (May 2022–December 2024).

	Examples of Indicators/Achievements
Donor recruitment and human milk collection	>600 human milk donation proposals (primarily via the HMB website) and followed by direct contact with potential donors >130 qualified donors, evenly split between internal donors, or mothers of hospitalized neonates who wish to donate their stored surplus milk after hospital discharge, and external or community-based donors >600 L of processed DHM (collected, pasteurized, and quality-checked) No shortage 100% batches consumed before the expiration date
DHM patients delivery	430 DHM patient recipients: 80% preterm and 20% critically ill neonates with other indications 100% of the DHM needs covered 100% parental acceptance (signed consent)
Nutritional and clinical outcomes *	80% breastfeeding bridging after DHM 47% reduction in NEC (2.6% vs. 4.9%); none required surgery (0% vs. 53%) 73% decrease in late onset sepsis (3.5% vs. 12.8%, alongside enhanced infection prevention measures) 30% decrease in median central catheter duration and 40% reduction in parenteral nutrition duration 2 days decrease in the length of hospital stay No critical incident related to DHM detected
Education and training	Participation in pre- and post-graduate medical and nursing educational programs Several master and thesis projects Around 200 requests/year for visits or observation periods from health professionals or students
Collaboration between CHUV and TIR	Recurring joint activities include: weekly operational meetings, two annual training days, biannual steering committee meetings, co-authored annual reports for public health authorities Additional collaborative efforts involve the development of shared documents, such as quality manuals and SOPs and joint participation in scientific events
Human milk and donation awareness	>50 media/public communications, >3000 annual website visits Positive feedback from donors, families, and health professionals. Regional public events (e.g., *World Human Milk Donation Day*, *World Breastfeeding Week*) National and international scientific meetings presentations Several awards and distinctions

* Comparisons of very preterm and very low birthweight patients between the 2 years after the HMB implementation (2023–2024) vs. the 2 years before (2020–2021), where applicable. NEC: necrotizing enterocolitis.

## Data Availability

The data presented in this study are available on request from the corresponding author (the data are not publicly available due to different IT safety formats but can be partially extracted on request).

## Supplementary Materials

The following supporting information can be downloaded at: https://www.mdpi.com/article/10.3390/nu17111765/s1, S1: Comprehensive description of *CHUV Lactarium* processes.

## Abbreviations

The following abbreviations are used in this manuscript:

ADLF	French human milk banks association (*Association des Lactariums de France*)
CHUV	Lausanne University Hospital (*Centre Hospitalier Universitaire Vaudois*)
DHM	donor human milk
DSAS	Department of Health and Social Action
EMBA	European Milk Banking Association
GAMBA	Global Alliance of Milk Banks and Associations
GMP	good manufacturing practices
HMB	human milk bank
MOM	mother’s own milk
MPHO	medical product of human origin
NEC	necrotizing enterocolitis
SoHO	substance of human origin
SOP	standard operating procedure
MNDS	Swiss Minimal Neonatal Data Set
TIR	Interregional Blood Transfusion of the Swiss Red Cross (*Transfusion InterRégionale*)
WHO	World Health Organization
