# Supplementary material for: Bridging the Milk Gap: Integrating a Human Milk Bank–Blood Bank Model to Reinforce Lactation Support and Neonatal Care
Source: Nutrients. 2025 May 23;17(11):1765. doi: 10.3390/nu17111765 (PMC12158243; doi:10.3390/nu17111765)
Supplement: Supplementary file 1 [file nutrients-17-01765-s001.zip › nutrients-3570964-supplementary.pdf]

nutrients

Bridging the milk gap: integrating a human milk bank-blood bank model to reinforce lactation support and neonatal care

Supplementary Materials

S1. Comprehensive description of CHUV Lactarium processes

Website and descriptive film: https://www.chuv.ch/fr/lactarium/

Donor eligibility, screening, selection and training

Human milk donations are voluntary, non-remunerated, and subject to stringent health and lifestyle criteria. Donors must provide informed consent prior to participation. Two categories of donors are recognized: "internal" and "external". Internal donors are lactating mothers who expressed and stored milk at the CHUV Infant Nutrition Centre during their child's hospitalization and later choose to donate a portion of their surplus milk after hospital discharge. External donors are breastfeeding women with a healthy, full-term infant who proactively decide to donate their surplus milk. Prospective donors contact the Lactarium directly through the website and undergo an initial interview and screening by phone with a Lactarium nurse. Preliminary eligibility is assessed based on general health, absence of toxic substance use, availability of surplus milk, proximity to the hospital, and confirmed motivation to donate. Candidates meeting these criteria are invited for an in-person consultation at the CHUV. During this first consultation, candidates complete a detailed medical questionnaire addressing their health, lifestyle, followed by serological testing for HIV, syphilis, hepatitis B and C, and, if indicated, HTLV-I/II and Chagas disease, which are analysed at the TIR laboratory. The test results, along with the completed questionnaire, are reviewed by the responsible physician. Upon approval, the Lactarium nurse schedules a second consultation with the donor. At this follow-up visit, donors receive comprehensive training and demonstration on hygiene protocols critical to milk expression and storage. This includes proper handling, cleaning, and sterilization of equipment to prevent contamination. This rigorous process aims to ensure the safety and quality of donated human milk while supporting donor confidence and adherence to best practices.

Donor human milk collection

External donors are provided with a comprehensive information guide along with the necessary materials to support safe and efficient milk donation. These materials include the loan of a double breast pump for the donation period, breast pump sets, bottles, barcoded identification labels, and thermometers to monitor the temperature of their home refrigerator and freezer. A temperature logger is placed on one of the first bottles collected to ensure the integrity of the cold chain from the initial expression. Donors are required to freeze their expressed milk as soon as possible (max 24 hours) after expression, and to label each bottle with date and time information. Milk collections at their home are scheduled by a transportation service every one to two weeks according to needs. Regular follow-up by phone is conducted by the Lactarium nurses to assess the donor's experience, providing support and guidance for optimal milk expression practices. Every three months, donors undergo routine serological testing at the hospital, and their breast pump sets are replaced to ensure proper hygiene.

Microbiological analyses, pasteurisation, storage and transportation

Upon arrival at the Lactarium processing facility of the blood bank TIR, each collected frozen donor human milk (DHM) bottle is systematically identified and logged into the software system with their associated information (notably expression date and

time, compliance of the delay between expression and freezing, visual compliance). Raw milk is stored at -30°C for maximum 3 months after expression until being processed. The milk is then thawed and 2 litres batches comprising pooled samples from 1-5 individual donors are prepared and pasteurised (Holder pasteurisation, 62.5°C, 30 min) in 100 mL bottles. Microbiological testing of the batches is performed both before and after pasteurisation. Milk batches that pass microbiological testing are stored at the blood bank in cold temperature room at -30°C. Conforming milk is then ordered and delivered to the hospital, where it is further stored (-20°C) and finally prepared and fortified when indicated, within 6 months after pasteurisation, by the Infant Nutrition Centre before to be provided within the next 24 hours to the recipient patients.

Should such microbiological analysis detect bacterial levels exceeding acceptable thresholds (e.g. >10^5 cfu/mL), donors of the batch are identified, and individual milk samples are analysed. In addition, the donor is contacted by the Lactarium nurse, retrained on proper hygienic practices, and provided with new materials to continue the donation process.

DHM delivery to recipients' patients

Indications and priority allocations are presented in the text. Before administration, parent's information and signed consent is required. Additional information targeted on milk kinship issues is available. DHM batch codes are scanned in recipient patient electronic files.

Disclaimer/Publisher's Note: The statements, opinions and data contained in all publications are solely those of the individual author(s) and contributor(s) and not of MDPI and/or the editor(s). MDPI and/or the editor(s) disclaim responsibility for any injury to people or property resulting from any ideas, methods, instructions or products referred to in the content.
